# Genetic and Epigenetic Changes in Plants in Response to Abiotic Stress

**DOI:** 10.3390/genes12101603

**Published:** 2021-10-12

**Authors:** Jong-Joo Cheong

**Affiliations:** Center for Food and Bioconvergence, Seoul National University, Seoul 08826, Korea; cheongjj@snu.ac.kr

The current global climate crisis has led to drought, high salinity, and abnormal temperatures (heat and cold), and is a serious threat to crop productivity. Plants have evolved various strategies to cope with such stressful conditions, undergoing physiological changes through genetic and epigenetic regulation of gene expression. Recent studies have revealed that these (epi)genetic changes are memorized and transmitted to new cells during vegetative growth and are even inherited by the next generation. The experimental studies and reviews published in this Special Issue focus on data and information obtained from (epi)genetic and (epi)genomic studies of plant responses to various abiotic stresses.

The original research article by Ni et al. [1] describes gene expression patterns altered under stress conditions in *Iris lactea* var. *chinensis*, a perennial herb halophyte exhibiting extremely high salinity and drought tolerance. The research team used the PacBio RSII sequencing platform to determine the transcriptomes differentially expressed in response to salinity stress, with NaCl treatment or drought stress imitated by polyethylene glycol (PEG) treatment. High NaCl and PEG concentrations resulted in 9266 and 8390 differentially expressed genes (DEGs), respectively. Of these, 3863 DEGs had the same expression pattern under both stresses, while 34 DEGs had different expression patterns. The transcriptome expression profiles obtained in this study provide a resource for further investigation of the mechanisms underlying the responses to multiple abiotic stresses in this species. Furthermore, the stress-related genes found here will be useful for future molecular breeding.

Exposure to drought or salt stress stimulates the production of reactive oxygen species (ROS), which induce a variety of stress responses in a plant. Excessive ROS accumulation causes severe damage to cell membranes and cellular organelles. The antioxidant enzyme ascorbate peroxidase (APX) catalyzes the formation of O_2_ and H_2_O from hydrogen peroxide (H_2_O_2_) to protect plants from oxidative damage. In a bioinformatic analysis, Leng et al. [2] identified 11 *APX* genes belonging to class I of the heme-containing peroxidase family in the *Populus trichocarpa* genome. Promoter sequence analysis revealed that the majority of the *PtrAPX* genes contained various phytohormone- and abiotic stress-related *cis*-elements. *PtrAPX* transcription was induced in response to drought, salinity, high ammonium concentrations, and exogenous abscisic acid (ABA) treatment. This finding provides a framework for further detailed analysis of the evolution and functions of *APX* genes in this species.

Two review articles in this *Special Issue* are devoted to epigenetic aspects of the molecular mechanism underlying plant responses to abiotic stress. When a gene is transcribed in response to stress signals, the chromatin around the gene transitions from a repressive to active state, enable RNA polymerase access. Chromatin remodeling is accompanied by changes in epigenetic marks, such as histone modification (acetylation and methylation), DNA methylation, and microRNA generation. Epigenetic marks may also provide a mechanistic basis for stress memory, which enables plants to respond more effectively and efficiently to recurring stresses. Miryeganeh [3] exhaustively reviewed the epigenetic mechanisms underlying plant tolerance to heat, cold, salt, and drought stresses. The author also described the epigenetic mechanisms that plants have evolved to adapt to fluctuating nutrient contents and UV-B stress. Furthermore, this review presents valuable information on recent advances in studies of the regulatory mechanisms underlying the intra- and inter-generational stress memory of abiotic stress responses in plants.

Li et al. [4] focused on histone acetylation, a ubiquitous epigenetic mark. In the chromatin of eukaryotic cells, genomic DNA wraps around histone octamers. The level of histone acetylation, which is regulated by histone acetyltransferases (HATs) and histone deacetylases (HDACs), determines whether the chromatin is open or closed, which controls access by RNA polymerases and DNA-binding regulatory proteins for transcriptional activation and repression, respectively. In their review, they summarized histone acetylation changes and functions of HATs and HDACs in drought resistance. HATs and HDACs function in many drought response signaling pathways, including the ABA, acetic acid, and jasmonic acid (JA) pathways, by changing the level of histone acetylation in their target genes. Other regulatory factors, such as transcription factors and cofactors (coactivators or corepressors), work cooperatively with HATs and HDACs to influence the levels of histone acetylation. The authors suggested many important studies that should be conducted to explore the detailed molecular mechanisms underlying the crosstalk between histone acetylation and other epigenetic marks.

In addition to histone acetylation and methylation, DNA methylation of cytosine bases in the nuclear genome is a conserved epigenetic mark that plays an important role in maintaining genome stability and regulating gene expression. Singh et al. [5] investigated the role of DNA methylation in response to heat stress in tomato plants (*Solanum lycopersicum*) by analyzing the DNA methylation-deficient mutant *Slddm1b*. They observed that the mutant was significantly less sensitive to heat stress than the non-mutant line. Under heat stress at the seedling stage, the mutant line had higher fruit- and seed-set rates and a higher survival rate. At the transcription level, they observed differences in the expression of heat stress-related genes, implying an altered response of the *ddm1b* mutant to heat stress. Based on these results, they propose further research on the contribution of DEGs to thermo-tolerance and their role in changing DNA methylation status. Since DNA methylation patterns may contribute to transgenerational inheritance, these observations provide a basis for improving crop heat stress tolerance.

The articles in this Special Issue provide valuable data and critical assessments of the molecular mechanisms underlying the genetic and epigenetic regulation of abiotic stress in plants. Most importantly, epigenetic modifications regulating gene expression and the ability to transfer acquired traits to the next generation constitute unique adaptation mechanisms for plants. Unlike traditional DNA sequence mutations, epigenetic mutations can be induced intentionally with considerably less time and effort, which makes them an attractive tool for breeding higher-quality crops that can withstand adverse climatic conditions.

## Data Availability

Not applicable.
